# Fatal drug poisonings in an industrial region of the Far North of Russia

**DOI:** 10.1192/j.eurpsy.2022.938

**Published:** 2022-09-01

**Authors:** A. Novikov, A. Gil

**Affiliations:** 1 Surgut city Clinical Psychiatric-and-Neurological Hospital, Chief Physician Office, Surgut, Russian Federation; 2 I.M. Sechenov First Moscow State Medical University (Sechenov University), Higher School Of Health Administration, Moscow, Russian Federation

**Keywords:** drug poisoning, Russia, overdose deaths, drugs

## Abstract

**Introduction:**

Drug poisonings is a continuous public health problem in Russia and worldwide.

**Objectives:**

The objective of the study was to provide characteristics of lethal drug poisonings in a northern Russian region.

**Methods:**

The data on deaths from drug poisoning that occurred between 2018 and September 2021, systematically collected by the Regional Center on the Organization of the Narcological and Psychiatric service of the Khanty-Mansi Autonomous Okrug of Russia, was analyzed.

**Results:**

Among 220 cases of fatal drug poisoning the overwhelming majority (90.4%) occurred among males. The number of cases increased annually from 27 in 2018 to 71 in 2020. The average age of death increased from 33.6 years in 2018 to 38.2 years in 2021. Over two thirds of deceased (70.9%) had complete secondary or vocational secondary level of education, almost one third (30.0%) were skilled workers, and slightly less than half (44.1%) were unemployed. The most common causes of death were methadone poisoning (34.5%), poisoning with other opioids (21.8%), other synthetic drugs (17.3%), other unspecified drugs (11.8%), and psychostimulants (10.0%). Alcohol intoxication was identified as a concomitant cause of death in every fourth case (26.0%), of which 98% were among males. Most often, alcohol was present in the blood at a concentration of 120 mg/ml and above. Every fourth deceased (23.6%) was registered with narcology health service for drug addiction.

**Conclusions:**

Fatal poisonings with narcotic drugs and psychotropic substances is a growing public health problem in a northern industrial region of Russia, which affects predominantly working-age males and requires comprehensive multisectoral response.

**Disclosure:**

No significant relationships.

